# Effect of Dietary Types on Feed Intakes, Growth Performance and Economic Benefit in Tibetan sheep and Yaks on the Qinghai-Tibet Plateau during Cold Season

**DOI:** 10.1371/journal.pone.0169187

**Published:** 2017-01-05

**Authors:** Tianwei Xu, Shixiao Xu, Linyong Hu, Na Zhao, Zhe Liu, Li Ma, Hongjin Liu, Xinquan Zhao

**Affiliations:** 1 Northwest Institute of Plateau Biology, Chinese Academy of Sciences, Xining, China; 2 University of Chinese Academy of Sciences, Beijing, China; 3 Key Laboratory of Adaptation and Evolution of Plateau Biota, Chinese Academy of Sciences, Xining, China; Sichuan University, CHINA

## Abstract

Pastoralists on the Tibetan alpine rangeland suffered great economic loss in cold season, due to serious live-weight loss of domestic livestock under traditional grazing management. This study aimed to evaluate the effect of dietary types (crude protein levels) on feed intakes, growth performance and economic returns of local Tibetan sheep and yaks during cold season. Twenty-four yearling Tibetan sheep (25.29±3.95 kg LW) and twenty two-year-old yaks (100.62±4.55 kg LW) with familiar body conditions were randomly assigned to four groups, fed oats hay (OH), oats silage (OS), total mixed ration (TMR) and traditionally grazed on the local cool-season pasture (TG), respectively, over a 135-day experiment. Daily dry matter intake was determined; all animals were weighed at the beginning and every 15 days of the 135-day experiment. Then, the total live-weight gain, average daily live-weight gain, gain rate, feed efficiency and net economic benefit were calculated. Results indicated that feed and nutrient intakes (DMI, DMI/kg LW, DMI/kg LW^0.75^ and CPI) of TMR, OH and OS were higher than TG (*P* < 0.05). Grazing animals suffered serious live-weight loss, while TMR, OS and OH significantly (*P* < 0.05) improved total live-weight gain and gain rate in both Tibetan sheep and yaks during the entire experiment. TMR worked better in animal performance and feed efficiency, obtained the highest breeding profit in both Tibetan sheep and yaks among four treatments (*P* < 0.05). When expressed on net economic benefit, TMR shared the highest net economic benefit in Tibetan sheep, OH shared the highest net economic benefit in yaks, but, no significant difference of net economic benefit in yaks fed TMR and OH diets was determined (*P* > 0.05). Results indicated that TMR was a reasonable diet in promoting feed intakes, animal performance, feed efficiency and economic returns in domestic livestock, which should be considered by local herdsmen to increase their breeding profit during cold season.

## Introduction

Qinghai-Tibet plateau (QTP) is an ecological functional zone and ecological security defense for China and even Asia due to its unique geographical location and climate characteristics [1,2]. Meanwhile, QTP is an important animal husbandry production zone, playing a vital role in improving local pastoralists’ livelihood [3].

Tibetan sheep (*Ovis aries*) and yaks (*Bos grunniens*) are the two major ruminant species, playing an increasingly important role on the Tibetan rangeland due to their excellent adaptability and production performance [4–7]. It is estimated with a population of about 13 million domestic yaks and 50 million Tibetan sheep are living on the QTP [8,9], providing local herdsmen with daily necessities like meat, milk, wool, skins, fuel and economic benefit [4,8,10]. Tibetan sheep and yaks breeding have laid a solid foundation of alpine pastoral economy and pastoralists’ livelihood on the QTP.

Under traditional grazing management, domestic livestock mainly lived on natural herbage of local pasture without feed supplementing [11]. Animals always suffered seasonal live-weight variations and viciously cycled in “alive in summer, strong in autumn, thin in winter, tired in spring”, due to seasonal fluctuations in herbage supply (biomass and nutrient content) and the contradiction between herbage supply and livestock’s requirement on the alpine rangeland [4,12,13]. When cold season came, grazing animals survived from inadequate herbage, low temperature and cold environment, which usually causing poor nutrition, health-related problem, low growth performance and even death of grazing livestock [10,13,14]. As a result, pastoralists suffered huge economic loss due to serious live-weight loss in grazing animals during cold season. Feed efficiency (total herbage intakes/total live-weight gain) and the off-take rate of livestock were quiet low under traditional grazing management [4,9,10,15]. More seriously, the vicious cycle was aggravated yearly due to over-stocking rate of livestock and irrational utilization of natural pasture on the QTP [16–18].

In order to improve the production efficiency of alpine pastoral grass-livestock husbandry, “Two-stage” management has been gradually adopted by local pastoralists in recent years. Domestic animals usually grazed on natural pasture during warm season (June to October), then, turned to warm-shed feeding during cold season (November to May). The newly mode significantly shortened livestock’s breeding cycle and increased economic returns for local herdsmen [9,19,20]. Dietary crude protein was an important factor affecting livestock’s growth performance and economic returns [21,22]. However, local pastoralists often choose/use dried hays (with low CP and high fiber contents) during warm-shed feeding period [9,23,24], which usually resulting in low growth performance and low economic returns. Here, we hypothesized that changing dietary processing methods (changing dietary CP levels) could affect animals’ feed intakes, growth performance, feed efficiency and economic returns during cold season on the QTP.

Therefore, this study aimed to (1) investigate the effect of dietary types (CP levels) on feed and nutrient intakes (DMI, DMI/kg LW, DMI/kg LW^0.75^, CPI and NDFI) in Tibetan sheep and yaks, (2) evaluate the growth performance in Tibetan sheep and yaks fed different diets, (3) account economic returns in Tibetan sheep and yaks fed different diets during cold season. Finally, we expected to find an optimal diet improving domestic livestock’s growth performance, increasing economic returns for pastoralists and alleviating grazing pressure of local cool-season pasture during cold season on the QTP.

## Materials and Methods

### Ethics Statement

During the experiment, all animals were cared for according to the Guide for the Care and Use of Laboratory Animals, the Ministry Science and Technology of People’s Republic of China (2002) [25]. The experimental design and procedures were approved by the Animal Ethic and Welfare Committee of the Northwest Institute of Plateau Biology, Chinese Academy of Sciences (NWIPB, CAS). All animals had free access to diet and water; they were well treated and no animal died during this study.

### Study Site

The field study was conducted in Qinghai Modern Prataculture Development Co., Ltd. (35°34′11″N, 100°46′45″E, altitude 3150 m) of Guinan County, Hainan Tibetan Autonomous Prefecture of Qinghai province, China. Climate here was dominated by plateau continental climate with short warm/growing season and long cold/non-growing season. The mean annual temperature was 3.1°C with extreme high 31.8°C in July and extreme low –29.2°C in January. The mean annual precipitation was 485.8 mm. Local cool-season pasture was alpine meadow dominated by *Kobresia humilis*, *Elymus nutans*, *Kobresia capillifolia*, *Stipa capillata*, *Poa annua and Carex atrofusca* et al.

### Animal Treatments and Experimental Diets

The study was established from December 15th of 2014 to May 2nd of 2015. Twenty four yearling Tibetan sheep (25.29±3.95 kg LW) and twenty two-year-old yaks (100.62±4.55 kg LW) with familiar body conditions were randomly assigned to four groups (n = 6 for Tibetan sheep and n = 5 for yaks), fed oats hay (OH), oats silage (OS), total mixed ration (TMR) and traditionally grazed on the local cool-season pasture (TG, treat as control). Before the experiment, warm-shed was sprayed using sodium hydroxide for disinfection; animals were fed deworming tablets to against internal parasites. A 14-day advance experiment was conducted to promote animals adapting to given diet and experimental environment. While the formal experiment began, animals in warm-shed were fed twice a day at 8:30 and 17:00, separately. Grazing animals were labeled and grazed with livestock crowd on the local cool-season pasture without feed supplementing, grazing activities usually lasted from 8:30 to 17:30 (about 9 hours per day), then entered shelter for overnight. All animals had freely access to multi-nutrient blocks and water over a 135-day experiment. Experimental animals were carefully observed for the occurrence of any health-related problems and records were taken throughout the entire experiment.

The oats hay was sown in early June 2014 and harvested in early October 2014 by a reaping machine, bounded into cubic bundles with approximately 19~22 kg, then stored in a dry and ventilated place. During the experiment, oats hay was chopped into 3~5 cm long pieces using a forage rubber to encourage animals’ feed intake. The oats silage was made by fresh oats herbage harvested in late September of 2014. The fresh oats herbage was cut into 5~8 cm long pieces by a harvester. Silage bacterial strain (made by Taiwan Yaxin Biotechnology Co. Ltd.) was added to fresh oats herbage with a recommended proportion of 100 mg for 5 ton silage, then stored in a silage pool and sealed for 70~90 days before feeding animals. Total mixed ration was fully mixed by oats hay, concentrate feeds, pre-mix, salt and water according to suitable proportions, using a mechanical agitator last for 45~55 mins to ensure nutritional equilibrium. Ingredients and nutrient composition of experiment diets was presented in Table 1.

**Table 1:** Ingredient and nutrient composition of experiment diets during the experiment.

Items	Treatments			
	TMR	Oats hay	Oats silage	Natural herbage
**Ingredients (%)**				
Oats hay	39.7			
Concentrate feeds ^a^	33.1			
Pre-mix ^b^	0.7			
Salt	0.4			
Water	26.1			
**Nutrient composition**				
DM, g/kg	684	881	571	921
CP, g/kg	103.1	56.9	84.0	51.0
EE, g/kg	27.4	21.2	23.1	19.0
NDF, g/kg	331.9	546.2	501.6	586.4
ADF, g/kg	139.6	317.3	285.2	368.3

DM is dry matter, CP is crude protein, EE is ether extract, NDF is neutral detergent fiber and ADF is acid detergent fiber.

^a^ Manufactured by Huanghexing Agriculture and Animal Husbandry Development Co., Ltd. contained maize, wheat, highland barley, bran and CaHPO_4_·2H_2_O et al. Nutritional level (%): DM≥86%, CP≥14%, CF≤8.8%, Salt = 0.4%, Ca = 0.8% and P = 0.6%.

^b^ Manufactured by Hehuangqingmu Animal Feeding S&T Development Co., Ltd., contained Forage vitamin, trace element, amino acid, Ca, P, Mg and NSP enzyme.

### Sampling, Measurement and Analyses

The amount of diets offered and residues of different diets were weighted and calculated every first ten days of every month to determine individual DMI. DMI of grazing animals was estimated by the formula that DMI = 0.028 × LW reported by Zhao [26].

Samples of different diets were collected and dried in forced-air oven at 60°C to constant weights (DM), then ground through a 1—mm sieve screen for further analyses. Total nitrogen (N) was measured according to Kjeldahl procedure and CP content was calculated by total N (CP = 6.25×N) [27]; Ether extract (EE) was determined by the Soxhlet system [27]; Neutral detergent fiber (NDF) and acid detergent fiber (ADF) were measured according to the methods described by Van Soest et al [28].

Animals were weighted by a special electronic balance (YIY-OCS-1T, made by Shanghai YiYu Electronics Technology Co., Ltd., with a sensitivity of 100 g) before morning feeding and grazing activities, at the beginning and every 15 days of the 135-day experiment. The total live-weight gain was calculated as the difference between final live-weight and initial live-weight; average daily live-weight gain (ADG) was defined as total live-weight gain divided by experiment time (day); gain rate was calculated by ratio of total live-weight gain to initial live-weight; feed efficiency was defined as ratio of total DM consume to total live-weight gain; breeding profit was calculated by the difference between the benefit of live-weight gain and total feed cost. Net economic benefit (NEB) was determined by the following equation,

$$\text{NEB}=\left(G_{w}\times P_{m}\right)∕\left(T_{e}\times DMI\times P_{d}\right)-1$$

Where *G*_*w*_ is total live-weight gain (kg), *P*_*m*_ is market unit price of live animals (¥/kg), *T*_*e*_ is the experiment time (day), DMI is daily DM intake (kg/d), *P*_*d*_ is unit price of given diet (¥/kg).

### Data Analysis

Data was initially processed by Microsoft Excel 2010 and presented as mean±S.E., one-way analysis of variance (ANOVA) with Duncan multi-comparison test was used to determine the effect of dietary types on feed intakes (DMI, DMI/kg LW, DMI/ kg LW^0.75^, CPI and NDFI), live-weight gain, gain rate, feed efficiency, breeding profit and net economic benefit. All analysis were achieved using soft package SPSS (Statistical Package for the Social Sciences, Version 20.0). Statistical significance differ when *P* < 0.05.

## Results

### Feed Intakes

The daily DM and nutrient intakes of animals fed different diets were presented in Table 2. There were significant differences of DMI, CPI and NDFI among TMR, OH, OS and TG diets in both Tibetan sheep and yaks (*P* < 0.05). DMI/kg LW was in the order TMR > OS > OH > TG in Tibetan sheep and TMR > OH > OS > TG in yaks. However, no significant difference of DMI/kg LW was determined between TMR and OH in both Tibetan sheep and yaks (*P* > 0.05). When expressed on metabolic LW (LW^0.75^) basis, DMI of TMR was higher than other three diets in both Tibetan sheep and yaks. Grazing animals (TG) shared the least DMI, DMI /kg LW, DMI/kg LW^0.75^ and CPI among four treatments (*P* < 0.05).

**Table 2:** Effect of dietary types on feed and nutrient intakes in Tibetan sheep and yaks (mean±S.E.).

Items	TMR	OH	OS	TG	SEM	*P*-value
**Tibetan sheep**						
DMI (kg/d)	1.41±0.03a	1.31±0.03b	1.22±0.03c	0.69±0.02d	0.02	< 0.001
DMI/kg LW (g/kg)	43.53±1.13ab	40.87±0.91b	43.75±1.36a	28.00c	0.67	< 0.001
DMI/kg LW^0.75^ (g/kg)	103.37±2.14a	97.10±2.13b	100.29±2.71ab	62.18±0.44c	1.55	< 0.001
CPI (g/d)	147.84±3.17a	74.47±1.89c	102.78±2.32b	35.18±0.93d	3.09	< 0.001
NDFI (g/d)	467.39±10.03c	714.88±18.11a	611.81±13.80b	404.52±10.70d	10.86	0.001
**Yaks**						
DMI (kg/d)	5.16±0.11a	3.77±0.09b	3.01±0.05c	2.63±0.05d	0.08	< 0.001
DMI/kg LW (g/kg)	32.83±0.78a	34.29±0.79a	29.03±0.59b	28.00b	0.36	< 0.001
DMI/kg LW^0.75^ (g/kg)	115.84±2.27a	110.89±2.40a	92.61±1.72b	86.99±0.46c	1.27	< 0.001
CPI (g/d)	532.51±10.85a	215.06±5.34b	253.21±3.89c	134.13±2.78d	11.10	< 0.001
NDFI (g/d)	1714.25±34.93b	2064.47±50.28a	1512.01±23.26c	1542.25±30.06c	23.87	< 0.001

Values that does not share the same letters are significantly (*P* < 0.05) different from each other.

### Growth Performance

**Fig 1:**
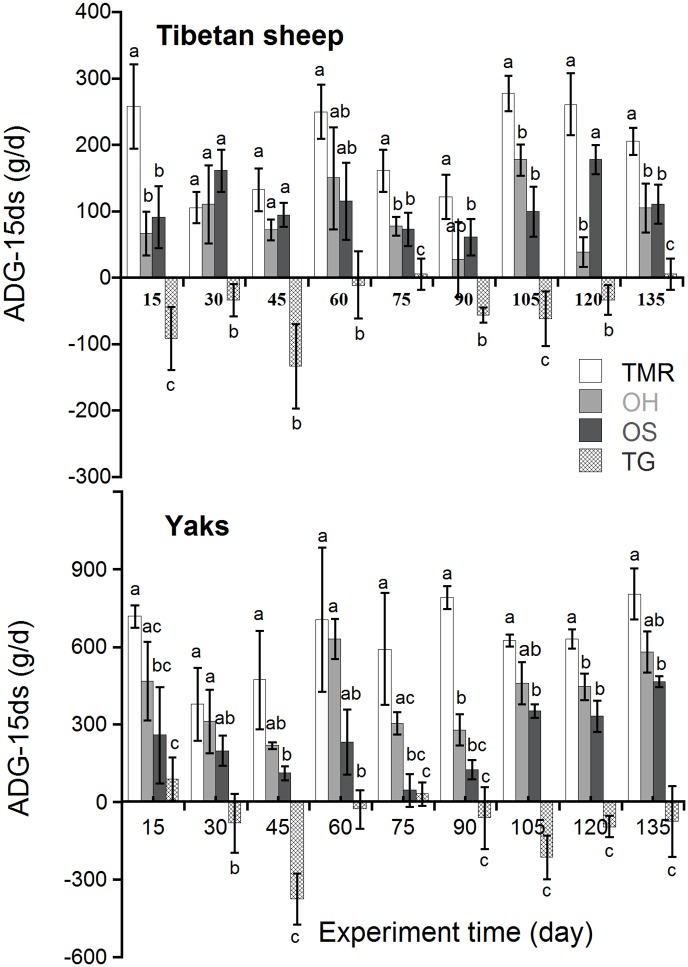
Changes of ADG-15ds in Tibetan sheep and yaks fed different diets during the experiment (mean±S.E.).

**Fig 2:**
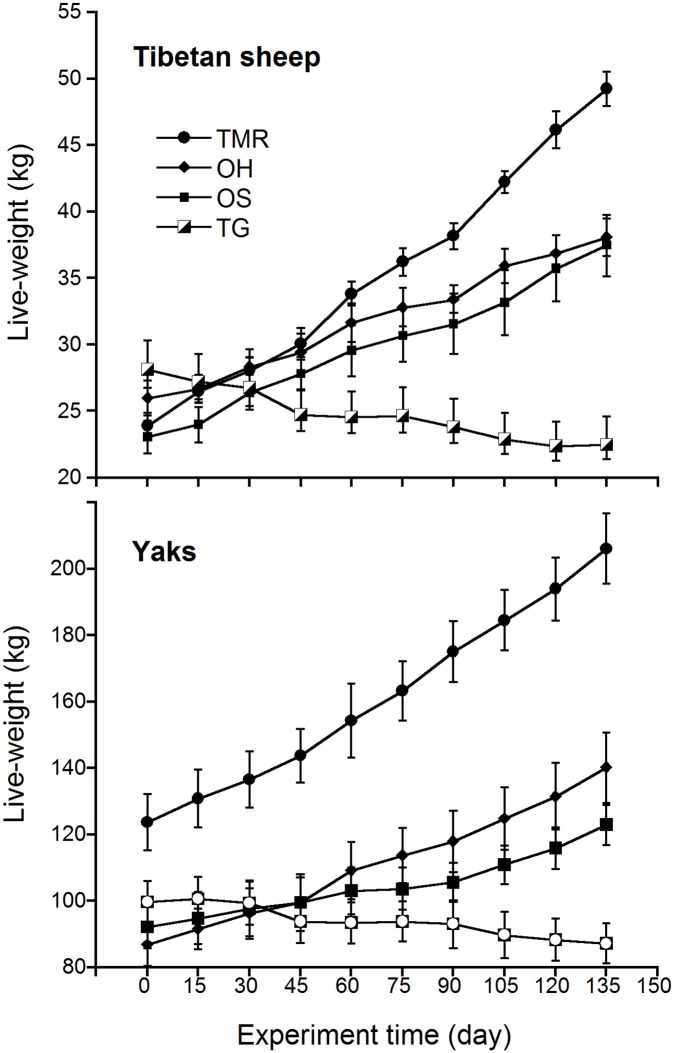
Live-weight variations of Tibetan sheep and yaks fed different diets during the experiment (mean±S.E.).

Average daily live-weight gain of every 15 days (ADG-15ds) during the experiment was presented in Fig 1. The figure indicated that ADG-15ds in TMR, OH and OS groups were better than TG group. Grazing animals suffered serious daily live-weight loss while warm-shed feeding animals shared great daily live-weight gain in most stages. No significant difference (*P* > 0.05) was determined of ADG-15ds in OH and OS groups (except 105–120 day period in Tibetan sheep). Fig 2 presented live-weight changes of animals fed different diets; which indicated that warm-shed feeding was more efficient in promoting growth performance in domestic animals during the entire experiment.

### Live-weight Gain and Feed Utilization

Grazing animals suffered serious live-weight loss (–5.66 kg, –20.54% in Tibetan sheep and –12.40 kg, –12.52% in yaks) under traditional grazing management (Table 3). By contrast, total live-weight gains of warm-shed feeding Tibetan sheep were 25.33 kg/sheep for TMR, 12.08 kg/sheep for OH and 14.33 kg/sheep for OS, which accounting for 107.45%, 47.94% and 61.92% of their initial live-weights, respectively. There was no significant difference of total live-weight gain, gain rate and ADG in Tibetan sheep feed OH and OS diets (*P* > 0.05). Total live-weight gains of warm-shed feeding yaks were 82.40 kg/yak for TMR, 53.32 kg/yak for OH and 30.70 kg/yak for OS, which accounting for 67.34%, 61.40% and 34.16% of their initial live-weights, respectively. Significant differences of total live-weight gain and ADG were found in yaks fed different diets (*P* < 0.05).

**Table 3:** Live-weight changes and feed efficiency in Tibetan sheep and yaks fed different diets during the experiment (mean±S.E.).

Items	TMR	OH	OS	TG	SEM	*P*-value
**Tibetan sheep**						
Initial LW (kg)	23.91±0.96ab	26.00±1.32ab	23.08±1.23b	28.17±2.16a	0.81	0.099
Final LW (kg)	49.25±1.29a	38.08±1.40b	37.41±2.27b	22.50±2.10c	2.16	< 0.001
Total LW gain (kg/sheep)	25.33±1.35a	12.08±1.40b	14.33±1.20b	–5.66±0.51c	2.38	< 0.001
ADG (g/sheep/d)	187.65±10.06a	89.51±10.41b	106.17±8.90b	–41.98±3.79c	17.64	< 0.001
Gain rate (%)	107.45±9.96a	47.94±7.32b	61.92±3.54b	–20.54±2.25c	9.99	< 0.001
Total DM consume (kg)	190.11	176.69	164.70	98.83		
Feed efficiency	7.65±0.38b	15.89±2.24a	11.92±1.04ab	–18.18±1.67c	2.86	< 0.001
**Yaks**						
Initial LW (kg)	123.70±8.45a	86.90±6.50b	92.20±6.44b	99.70±6.35b	4.55	0.009
Final LW (kg)	206.10±10.64a	140.22±10.61b	122.90±5.99b	87.30±5.95c	10.65	< 0.001
Total LW gain (kg/yak)	82.40±3.05a	53.32±4.66b	30.70±1.91c	–12.40±1.13d	8.06	< 0.001
ADG (g/yak/d)	610.37±22.60a	394.96±34.57b	227.41±14.18c	–91.85±8.39d	59.73	< 0.001
Gain rate (%)	67.34±3.42a	61.40±3.29 a	34.16±3.59 b	–12.52±1.11c	0.07	< 0.001
Total DM consume (kg)	696.6	510.3	406.3	329.4		
Feed efficiency	8.50±0.32b	9.90±0.95ab	13.45±0.86a	–27.57±2.77c	3.88	< 0.001

Values that does not share the same letters are significantly (*P* < 0.05) different from each other.

**Fig 3:**
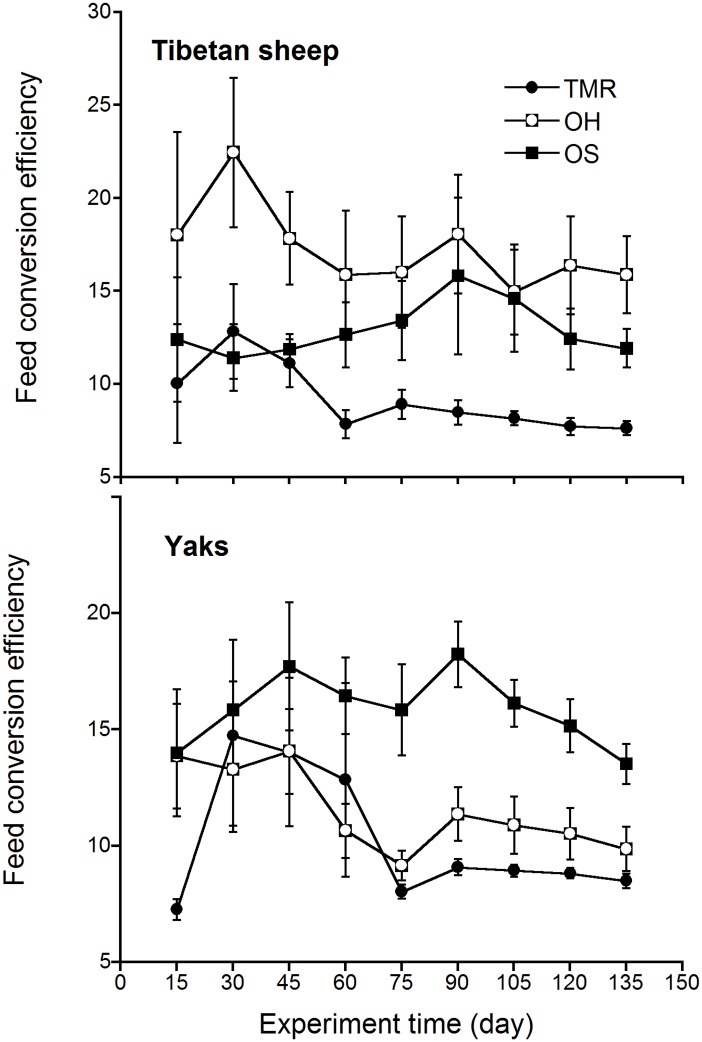
Changes of feed efficiency in Tibetan sheep and yaks fed different diets during the experiment (mean±S.E.).

Feed efficiency was an important index reflecting digestion and absorption efficiency of a given dietary [4,29]. In this study, feed efficiency of Tibetan sheep fed TMR and OS maintained a relative efficient level after 45^th^ day of the experiment (Fig 3), OH was the least efficient in feed conversion among three given diets. There was a trend that dietary with higher CP level was better in feed efficiency in Tibetan sheep. Feed efficiency of yaks fed TMR and OH maintained a relative efficient level after 60^th^ day of the experiment, OS was the least efficient among three given diets. Overall results indicated that feed efficiency of TMR (7.65 for Tibetan sheep and 8.50 for yaks) was better as compared to OH and OS (Table 3).

### Economic Benefit

Breeding profits of Tibetan sheep fed different diets were 205.01 ¥/sheep for TMR, 16.18 ¥/sheep for OH, 136.30 ¥/sheep for OH and –119.00 ¥/sheep for TG, respectively. Net economic benefits were 0.63 for TMR, 0.05 for OH and 0.58 for OS (Table 4), respectively. There was no significant difference of breeding profit and economic benefit in Tibetan sheep fed OS and TMR (*P* > 0.05). Breeding profits of yaks fed different diets were 1016.71 ¥/yak for TMR, 734.14 ¥/yak for OH, 248.32 ¥/yak for OS and –333.31 ¥/yak for TG, respectively. Net economic benefits were 0.85 for TMR, 1.04 for OH and 0.43 for OS. Significant difference of breeding profit was determined among yaks fed TMR, OH and OS (*P* < 0.05); however, there was no significant difference of net economic benefit (*P* > 0.05) in yaks fed OH and TMR diets. TMR was a proper diet in increasing the breeding profits for local herdsmen during cold season.

**Table 4:** Economic returns in Tibetan sheep and yaks fed different diets during the experiment.

Items	TMR	OH	OS	TG	SEM	*P*-value
**Tibetan sheep**						
Feed price(¥/kg DM)	1.72	1.37	1.42	—		
Feed consume (kg DM)	190.11	176.69	164.70	98.83		
Total feed cost(¥)	326.99	242.07	233.87	—		
Benefit of LW gain(¥)	532.00±28.52a	253.75±29.5b	301.00±25.23b	–119.00±10.73c	50.02	< 0.001
Breeding profit(¥/sheep)	205.01±28.52a	11.68±29.50b	136.30±25.23a	–119.00±10.73c	28.24	< 0.001
Profit over TG (¥/sheep)	324.01±36.47a	130.68±36.54b	255.30±24.69a	—	26.41	0.003
Net economic benefit	0.63±0.09a	0.05±0.12b	0.58±0.11a	—	0.086	0.003
**Yaks**						
Feed consume (kg DM)	696.60	510.30	406.30	329.41		
Total feed cost(¥)	1198.15	699.11	576.95	—		
Benefit of LW gain(¥)	2214.91±82.02a	1433.24±125.47b	825.21±51.46c	–333.31±30.47 d	216.7	< 0.001
Breeding profit (¥/yak)	1016.71±82.02a	734.14±125.47 b	248.32±51.46c	–333.31±30.47d	123.3	< 0.001
Profit over TG (¥/yak)	1349.71±82.02a	1067.14±125.47b	581.32±51.46c	—	159.7	< 0.001
Net economic benefit	0.85±0.07a	1.04±0.18a	0.43±0.09b	—	0.095	0.011

Values that does not share the same letters are significantly (*P* < 0.05) different from each other.

## Discussion

Tibetan sheep and domestic yaks are important resources for herdsmen who live in the alpine pastoral area as providing them daily necessities and economic income. However, under traditional grazing management, production efficiency of alpine pastoral husbandry and feed efficiency was quiet low due to irrational grazing-management regime and environmental factors [9,10]. In addition, natural grassland degradation became increasingly serious due to over-stocking and irrational utilization of nature pasture, which hampered the sustainable development of alpine pastoral grass-livestock husbandry on the QTP [9,16].

We expected to find a proper diet promoting domestic animals’ growth performance, increasing local herdsmen’s breeding profit and alleviating grazing pressure of local cool-season pasture. Our results indicated that total mixed ration was an appropriate diet in improving feed intakes, growth performance, feed efficiency and economic returns in both Tibetan sheep and yaks, which should be considered by local herdsmen to improve their breeding profit during cold season.

### Feed and Nutrient Intakes

Daily DMI was in the order TMR > OH > OS > TG and CPI was in the order TMR > OS > OH > TG in both Tibetan sheep and yaks. When expressed on LW and metabolic LW (LW^0.75^) basis, DMI of TMR, OH and OS were significantly increased as compared to TG. The NDFI was in the order OH > OS > TMR > TG for Tibetan sheep and OH > TMR > TG > OS for yaks. Grazing animals shared the least DM and nutrient intakes during the experiment (Table 2).

DMI was an important index in ruminant nutrition, which was affected by dietary nutrient levels, live-weight, health condition, production level, management and temperature etc. [30–32]. Under traditional grazing management, Tibetan sheep and yaks grazed on standing dormant herbage which was insufficient in biomass, crude protein and digestible carbohydrate contents [4,33,34]. With cold-season extension, standing herbage biomass and nutrient content decreased dramatically, leading to herbage shortage for grazing animals (November to May). As a result, grazing animals suffered inadequate DMI and inferior nutrients which could not meet their daily requirement [10,35].

Warm-shed feeding animals were offered diets with high CP contents; animals could freely seek forage to meet their daily DM requirement. In this study, a trend that higher dietary CP content encourage animals’ DM and nutrient intakes was found, which was agreed with Phengvichith V in goats [36], Negesse T in growing male Saanen kids [37], Antti N in male goat kids [38], and Li in Liaoning Cashmere Goat [39]. Herbage silage could maintain a relative higher CP content of fresh oats as compared to dried oats hay [40,41]. TMR was an advanced technology produce dietary with balanced nutrition and good palatability [42], promoting domestic animals’ DM intakes and feed efficiency [43–45]. In this study, TMR worked best in promoting feed and nutrient intakes in both Tibetan sheep and yaks (Table 2); the reasons could be related to its palatability, higher CP content, balanced nutrition [46], and low rumen fill effect of TMR [47]. During current experiment, TMR was full mixed from oats hay, concentrate feeds, pre-mix, salt and water. Reasonable oats hay length (2–4 cm), moderate concentrate feed proportion (42.2%), long mixed time (45–55 min) higher CP content (10.31%) and proper feed moisture (31.6%) ensured nutritional equilibrium and good palatability of TMR diet as compared to OH, OS and standing herbage. As a result, TMR diet avoided partial eclipse and malnutrition of warm-shed feeding animals and encouraged animals’ DM and nutrient intakes. In addition, the difference of temperature inside and outside the warm-shed was also an important factor affecting animals’ feed intake [48].

### Growth Performance and Feed Utilization

Warm-shed feeding animals shared great daily live-weight gains while grazing animals suffered serious daily live-weight loss in most stages during the experiment. Total live-weight gain was in the order TMR > OS > OH > TG in Tibetan sheep and TMR > OH > OS > TG in yaks (Table 4), no significant difference of total live-weight gain was determined in Tibetan sheep fed OH and OS diets (*P* > 0.05).

Under traditional grazing management, standing herbage biomass and nutrient content of local pasture decreased sharply during cold season, animals need to move more distance to seek herbage to meet their daily DM requirements [33]. Grazing animals consumed more energy and fat to oppose cold stress and maintain grazing activities as compared to warm-shed feeding animals, resulting in low growth performance, health-related problems and even death [4,9,10,14]. Tibetan sheep could lose 12.4%~43.7% of their initial live-weights [49], domestic yak could lose 25%~30% of their initial live-weights during cold season [35], which accounted for 80%~120% their live-weights that gained during last warm season [13]. In this study, grazing Tibetan sheep lost 20.54% and grazing yaks lost 12.52% of their initial live-weights during cold season, which meant heavy economic loss to local herdsmen.

Under warm-shed feeding, diets were rich in CP content (Table 1); air temperature was higher inside the warm-shed. Animals could freely seek diets, multi-nutrient block and fresh water to meet their growth requirements. In addition, warm-shed feeding reduced animal activities and energy consume as compared to traditional grazing, resulting in better growth performance of animals during harsh cold season. We found that total live-weight gain of animals fed higher CP diet was better (*P* < 0.05), which was agree with Dong in yaks [4], and Mulligan F in cattle [50]. This may be related to more DM and nutrient intakes of TMR as compared to other three diets [51]. The total live-weight gain of yaks fed oats silage (OS) was not met to our expectations, perhaps due to yak rumen could not adapt well to single oats silage diet, further study need to be conducted to evaluate the associated effect of concentrate and oats silage in yaks, relevant study in yaks was still sparse nowadays.

Feed efficiency was in the order of TMR > OS > OH > TG for Tibetan sheep and TMR > OH > OS > TG for yaks. Dietary with higher CP obtained better feed efficiency and animals’ growth performance, which was agreed with Chen in yaks [47], and Li in Dorper × Thin-tailed han crossbred weaning lambs [52]. The reasonable forage/concentrate ratio, stable feed value and proper feed moisture of TMR diet could increase rumen microbial activity and protein synthesis rate, maintaining normal fermentation, digestion, absorption and metabolic activities of domestic livestock, resulting in better growth performance and feed efficiency.

### Economic Benefit

Breeding profit for Tibetan sheep was in the order TMR > OS > OH > TG. Grazing Tibetan sheep suffered –119.00 ¥/sheep loss mainly due to serious live-weight loss during cold season. Net economic benefits were 0.63:1, 0.05:1 and 0.58:1 for TMR, OH and OS, respectively. Sheep fed OH gained much live-weight but shared low breeding profit and net economic benefit than Tibetan sheep fed OS. This could be explained that the benefit of live-weight gain of sheep fed OH was about flat with total feed cost (Table 4). Higher CP dietary contributed to high net economic benefit in Tibetan sheep was determined in current study.

When came to yaks, breeding profit was in the order TMR > OH > OS > TG. Grazing yaks suffered the lowest economic returns among four treatments, yaks with higher DMI (TMR > OH > OS > TG, Table 2) shared better live-weight gain, resulting in higher benefit of live-weight gain and breeding profit. Net economic benefits were 0.84:1, 1.04:1 and 0.43:1 for TMR, OH and OS, respectively. No significant difference of net economic benefit in yaks fed OH and TMR was determined (*P* > 0.05). The difference of net economic benefit was possibly attributed to different growth performance of yaks fed different diets. Yaks fed OH shared the highest net economic benefit. Dietary with moderate CP content shared a reasonable economic benefit was expected, which was agreed with Dong in yaks [4]. Dietary with moderate concentrate feed level (TMR) was also acceptable for its efficiency in improving growth performance and breeding profit in domestic animals. Other report also obtained that diet with moderate concentrate feed produce reasonable economic returns [35].

## Conclusion

Here, we evaluated the effect of dietary types on feed intakes, animal performance and economic benefit in domestic livestock during cold season on the QTP. Under traditional grazing management, domestic yaks and Tibetan sheep suffered serious live-weight loss, while, TMR, OS and OH significantly improved animals’ feed intakes, live-weight gain, feed efficiency and economic returns. Higher CP dietary obtained better growth performance, feed efficiency and breeding profit was determined in current study. TMR worked better in improving feed intakes, animal performance and economic returns, mainly due to its higher dietary CP content, nutritional equilibrium, proper feed moisture and good palatability, which should be considered by local herdsmen for promoting animals performance and increasing their breeding profit during cold season on the QTP.

## Supporting Information

S1 Data Live-weight changes in Tibetan sheep and yaks during the experiment. (XLSX)

S2 Data Average daily live-weight gains of every 15 days (ADG-15ds) in Tibetan sheep and yaks during the experiment. (XLSX)
